# Structural Basis for Long Residence Time c-Src Antagonist: Insights from Molecular Dynamics Simulations

**DOI:** 10.3390/ijms251910477

**Published:** 2024-09-28

**Authors:** Haiyang Zhong, Zhengshuo Zhang, Mengdan Chen, Yue Chen, Can Yang, Yunsheng Xue, Pei Xu, Hongli Liu

**Affiliations:** 1 Jiangsu Key Laboratory of New Drug Research and Clinical Pharmacy, Xuzhou Medical University, 209 Tongshan Road, Xuzhou 221004, China; 2 College of Pharmaceutical Sciences, Zhejiang University, Hangzhou 310058, China

**Keywords:** molecular dynamics simulations, c-Src, dissociation, residence time

## Abstract

c-Src is involved in multiple signaling pathways and serves as a critical target in various cancers. Growing evidence suggests that prolonging a drug’s residence time (RT) can enhance its efficacy and selectivity. Thus, the development of c-Src antagonists with longer residence time could potentially improve therapeutic outcomes. In this study, we employed molecular dynamics simulations to explore the binding modes and dissociation processes of c-Src with antagonists characterized by either long or short RTs. Our results reveal that the long RT compound DAS-DFGO-I (DFGO) occupies an allosteric site, forming hydrogen bonds with residues E310 and D404 and engaging in hydrophobic interactions with residues such as L322 and V377. These interactions significantly contribute to the long RT of DFGO. However, the hydrogen bonds between the amide group of DFGO and residues E310 and D404 are unstable. Substituting the amide group with a sulfonamide yielded a new compound, DFOGS, which exhibited more stable hydrogen bonds with E310 and D404, thereby increasing its binding stability with c-Src. These results provide theoretical guidance for the rational design of long residence time c-Src inhibitors to improve selectivity and efficacy.

## 1. Introduction

The proto-oncogene c-Src, a non-receptor tyrosine kinase, is central to many cellular processes, including cell survival, migration, and proliferation [1,2]. Dysregulation of c-Src has been implicated in multiple cancer types [3,4], highlighting its potential as a valuable target for drug discovery. Recent studies underscore its critical role not only in driving oncogenic behaviors but also in contributing to the development and persistence of resistance to various chemotherapeutic and targeted drugs [5,6,7,8], reinforcing the importance of c-Src as a critical therapeutic target.

c-Src is a member of the Src kinase family, which comprises nine proteins: c-Src, Fyn, Yes, Lck, Lyn, Hck, Fgr, Blk, and Yrk [9]. The high sequence similarity among these kinases, particularly within the highly conserved ATP-binding pocket, presents a significant challenge in developing highly selective inhibitors for c-Src. Currently, marketed c-Src inhibitors, such as dasatinib, bosutinib, and ponatinib, are ATP-competitive binding inhibitors characterized by strong binding affinity, with IC_50_ values in the nanomolar to the picomolar range [10,11,12]. However, these inhibitors also exhibit multi-target activity, resulting in low selectivity, which can diminish efficacy and lead to off-target toxicities. For instance, dasatinib and bosutinib not only inhibit c-Src but also kinases, such as Abl, causing adverse effects like neutropenia, thrombocytopenia, and anemia due to their lack of selectivity [13,14]. Therefore, enhancing the selectivity of inhibitors is crucial for improving efficacy and minimizing off-target side effects.

Traditional drug discovery has primarily focused on enhancing the equilibrium binding affinity of drug candidates to their target proteins [15,16], exemplified by the inhibition constant K_i_, which reflects the strength of a drug’s inhibition; a lower K_i_ indicates a stronger binding affinity [17]. However, binding affinity alone does not fully capture the drug’s interaction with its receptor in vivo, and many high-affinity compounds exhibit poor efficacy in clinical settings. Increasing evidence indicates that the residence time (RT) of drug-target binding, defined as the reciprocal of the dissociation rate constant (k_off_), plays a crucial role in determining drug efficacy and selectivity [18,19,20]. RT represents the duration a drug remains bound to its target before dissociating, significantly influencing its overall duration of action [21,22]. For example, Uitdehaag et al. investigated a series of threonine tyrosine kinase (TTK) antagonists with varying residence times and their relationship to cellular activity. The results indicated that the residence time of TTK inhibitors correlates with antiproliferative activity, with antagonists exhibiting longer residence times linked to increased antiproliferative efficacy [18]. Similarly, Wood et al. demonstrated that lapatinib, a tyrosine kinase inhibitor used in cancer, is a potent inhibitor of epidermal growth factor receptor (FGFR). Although lapatinib exhibits lower affinity for EGFR compared to compound Tarceva (K_i_ value of 3 nM and 0.4 nM, respectively), its residence time exceeds that of Tarceva by over 30 times (40 min vs. <14 min), leading to a more prolonged inhibitory effect on tumor cells [23]. Moreover, Zhang et al. developed a series of vasopressin V_2_R antagonists with varying K_i_ values and residence times and studied their impact on autosomal dominant polycystic kidney disease. Their results revealed that inhibitory effects on renal cyst growth correlated with the RT of the compounds rather than their affinity [19]. Additionally, the cyclin-dependent kinase 8 associated with cyclin C (CDK8/CycC) and discoidin domain receptors 1 (DDR1) are high-affinity targes of sorafenib, with similar selectivity when considering binding affinity alone (K_d_ values of 30 nM for CDK8/CycC and 72 nM for DDR1) [24]. However, sorafenib dissociates significantly more slowly from CDK8/CycC than DDR1, resulting in prolonged binding to CDK8/CycC. In vivo studies show that after 7 h, sorafenib no longer inhibits the rapidly dissociating DDR1, while 90% of CDK8/CycC activity remains inhibited. This finding suggests that compounds with slower dissociation rates (longer RT) exhibit higher target selectivity and improved efficacy. Similarly, Guo et al. assessed the binding kinetics of adenosine receptor (AR) antagonists across three AR subtypes (A_1_R, A_2A_R, and A_3_R) using competitive binding assays [25]. They found that although compounds xanthine amine congener (XAC) and 2,6-diphenyl-8-propyl-9H-purine (LUF5964) were non-selective in terms of binding affinity, they displayed high kinetic selectivity for A_1_R and A_3_R, respectively. These findings underscore the significance of drug-target binding kinetics in determining drug selectivity and efficacy.

With advancements in structural biology and computational power, molecular dynamics (MD) simulations have become widely utilized to analyze drug binding and dissociation mechanisms, thereby guiding drug design [26,27,28]. MD simulations provide detailed structural and energetic information over time and can readily replicate various experimental conditions. Zhong et al. recently employed conventional MD with umbrella sampling methods to elucidate the binding mode and dissociation processes of BRD4 inhibitors, revealing the crucial role of water bridges in ligand activity. This insight informed the optimization of lead compounds, ultimately successfully identifying highly active BRD4 inhibitors [28]. Similarly, Xu et al. explored the selectivity mechanisms of norepinephrine at β_1_AR and β_2_AR through metadynamics, discovering that charge differences along the binding pathway were the primary factors contributing to selectivity [29]. Liu et al. investigated the spontaneous binding process of Tolvaptan using Gaussian accelerated MD, identifying key residues involved in the binding pathway. They validated these critical residues through site-directed mutagenesis experiments, demonstrating the accuracy and reliability of their simulation results [30]. These studies underscore the significant advantages of MD simulations in guiding drug design.

**Figure 1:**
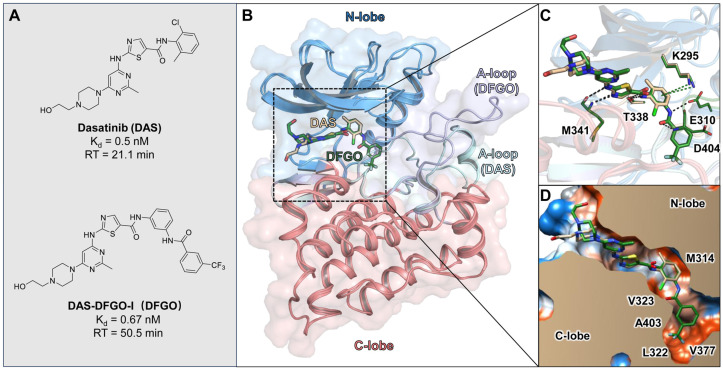
Crystal structure binding modes of DAS and DFGO with the c-Src: (**A**) Structures of DAS and DFGO, along with their thermodynamic and kinetic parameters. (**B**) Superimposition of DAS (wheat stick) and DFGO (green stick) with c-Src. (**C**) Comparison of key interactions in the DAS and DFOG crystal structures, with black dashed lines representing hydrogen bonds and green dashed lines indicating π−π stacking. (**D**) Comparison of hydrophobic interaction in the DAS and DFGO crystal structures, with polar regions depicted in blue and hydrophobic regions shown in orange.

To design highly selective c-Src inhibitors, targeting compounds with long residence times is a promising strategy. However, the interaction mechanisms between c-Src and long RT compounds remain poorly understood. In this study, we focus on two c-Src inhibitors reported by Kwarcinski et al.—dasatinib and DAS−DFGO−I (here abbreviated as DAS and DFGO, respectively)—which exhibit similar binding affinities but differ in residence times (Figure 1A) [31]. Utilizing Gaussian accelerated molecular dynamics simulations, we aim to elucidate the structural basis of interactions between c-Src and these compounds, identifying the key structural features that influence these interactions across varying residence times. This research aims to establish a theoretical framework for designing c-Src inhibitors with prolonged residence times, offering the potential to contribute to the development of more effective cancer therapies.

## 2. Results and Discussion

### 2.1. Binding Site Analysis

Compounds DAS and DFGO share the same core structure, with the primary difference being that DFGO extends the structure of DAS by adding a trifluoromethyl-substituted benzene ring via an amide bond (Figure 1A). Despite their similar binding affinity, this structural modification results in a twofold increase in the residence times for DFGO, which has a longer residence time (RT = 50.5 min). The binding mode observed in the crystal structure shows that, compared to DAS, the trifluoromethylbenzyl group of DFGO occupies an allosteric pocket (Figure 1B), forming hydrogen bonds with E310 and D404 (Figure 1C), and hydrophobic interactions with multiple residues, such as L322 and V377 (Figure 1D). The allosteric binding may be one of the factors contributing to DFGO’s prolonged residence time, as the additional interactions within this pocket likely stabilize the ligand–protein complex. Previous studies have similarly suggested that the occupation of allosteric pockets can enhance the binding duration of inhibitors. The combination of hydrogen bonding and hydrophobic interactions in this pocket may create a more rigid binding environment, slowing dissociation and increasing residence time, which is a key parameter in achieving sustained therapeutic effects [32,33,34]. Intriguingly, despite the additional hydrogen bond interaction, DFGO does not exhibit a significantly higher binding affinity compared to DAS. This observation suggests that the hydrogen bonds formed by DFGO with E310 and D404 play a more crucial role in the drug dissociation process rather than merely enhancing binding affinity. To investigate the underlying reasons for this difference, we conducted molecular dynamics (MD) simulations to characterize the dynamics behavior of these two ligands. 

**Figure 2:**
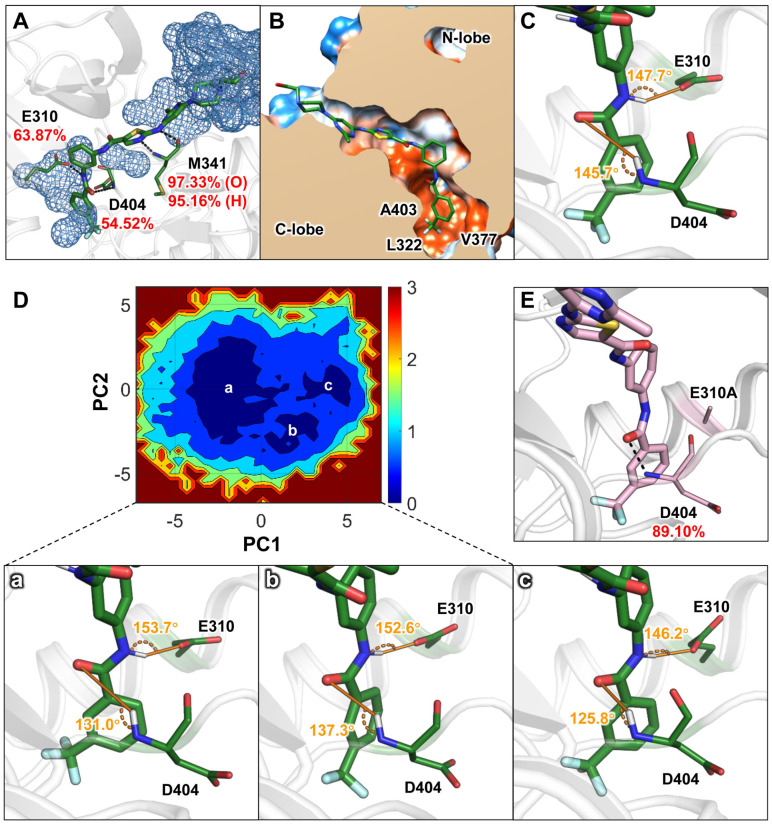
Structural characteristics of the ligand in the ATP binding pocket as revealed by conventional molecular dynamics (cMD) simulations: (**A**) Water distribution with 5 Å of DFGO and the proportion of hydrogen bonds formed with E310, M341, and D404. Black dashed lines represent hydrogen bonds. (**B**) Hydrophobic interaction map of DFGO. Polar regions are depicted in blue, and hydrophobic regions are shown in orange. (**C**) Angles of hydrogen bonds formed between DFGO and residues E310 and D404, with the donor−acceptor distance of the hydrogen bond between DFGO and D404 measured at 4.6 Å. (**D**) Free energy landscape constructed by the cMD and the represent conformations of a, b, and c, where the donor–acceptor distances of the hydrogen bonds between DFGO and D404 are 4.6 Å, 3.9 Å, and 3.3 Å, respectively. (**E**) Proportion of hydrogen bond formation between D404 and DFGO after E310 is mutated to A310 in the DFGO system.

To further elucidate the interaction characteristics between c-Src and small-molecule compounds with differing residence times, we conducted three parallel molecular dynamics simulations to explore the structural features of ligand interactions within the c-Src binding pocket. Root means square deviation (RMSD) analysis (Figure S1A,B) showed that the RMSD values for both systems converged, and the final 100 ns of the trajectory were used for subsequent analyses. Binding free energies of the ligands to c-Src were calculated using the molecular mechanics/generalized Born surface area (MM/GBSA) method. As detailed in Table 1, the binding free energies (∆G) for DFGO and DAS were similar, at −30.33 and −29.60 kcal/mol, respectively, which is consistent with their similar activities. However, compared to the DAS system, DFGO exhibited a 3.5–fold increase in polar solvation energy (ΔG_GB_), indicating a significant desolvation barrier. Further analysis of hydrogen bonds revealed that those formed by DFGO with D404 and E310 had relatively low occupancy rates of 54.52% and 63.87%, respectively (Figure 2A), suggesting these hydrogen bonds were unstable. Specifically, hydrogen bond between DFGO and E310 was exposed to the aqueous environment (Figure 2A), which would significantly reduce its stability [35]. Conversely, the hydrogen bond between DFGO and D404 was situated in a hydrophobic environment (Figure 2B), where hydrogen bonds are theoretically more stable [35]. However, the hydrogen bond angle between DFGO and D404 was less than 150°, and the donor–acceptor distance exceeded 2.9 Å (Figure 2C). Typically, hydrogen bond angles greater than 150° and the hydrogen bonds between an amide carbonyl and an amine around 2.9 Å are generally considered more favorable for hydrogen bond stability [36,37,38]. This suboptimal angle likely contributes to the reduced stability of the DFGO–D404 hydrogen bond.

To further investigate the instability of the two hydrogen bonds in the DFGO systems, principal component analysis (PCA) was performed on the trajectories, and a free energy landscape was constructed based on the results. As the eigenvector, we selected the RMSD value of the nonhydrogen atoms of DFGO, E310, and D404. As shown in Figure 2D, three primary energy wells were identified, from which representative conformations were extracted from each well. The results revealed that the hydrogen bond angle between D404 and DFGO in all three representative structures was consistently less than 150° (Figure 2D), corroborating the findings from hydrogen bond analysis. Additionally, energy decomposition analysis indicated that in all simulated trajectories of the DFGO system, E310 contributed positively (Table S1), suggesting a destabilizing effect on the ligand’s binding to c-Src. These results led us to hypothesize that the inherent instability of the hydrogen bond between E310 and DFGO may influence the conformational dynamics of the ligand, thereby reducing the stability of the hydrogen bond formed between DFGO and D404.

To test this hypothesis, we mutated residue E310 to A310 and conducted a 100 ns MD simulation. The results demonstrated that the energy contribution of A310 became negative (Table 1), and the occupancy of the hydrogen bond between DFGO and D404 increased from 54.52% to 89.10% (Figure 2E), further confirming that E310 affects the stability of the hydrogen bond formed between DFGO and D404. In summary, both hydrogen bonds formed by DFGO with E310 and D404 are unstable, and E310 appears to influence the stability of the hydrogen bond formed between DFGO and D404.

### 2.2. Dissociation Pathway Analysis

Dror et al. revealed that, in addition to the residues within the ligand–binding pocket, critical residues along the ligand’s entry pathway are also essential for effective ligand-target binding [26]. Additionally, Guo et al. demonstrated that mutations in key residues along the ligand dissociation pathway can profoundly affect the ligand’s dissociation rate [39]. These insights suggest that targeting critical residues along the dissociation pathway of small molecules could enhance the selectivity of c-Src inhibitors, particularly within the highly conserved ATP binding pocket.

**Figure 3:**
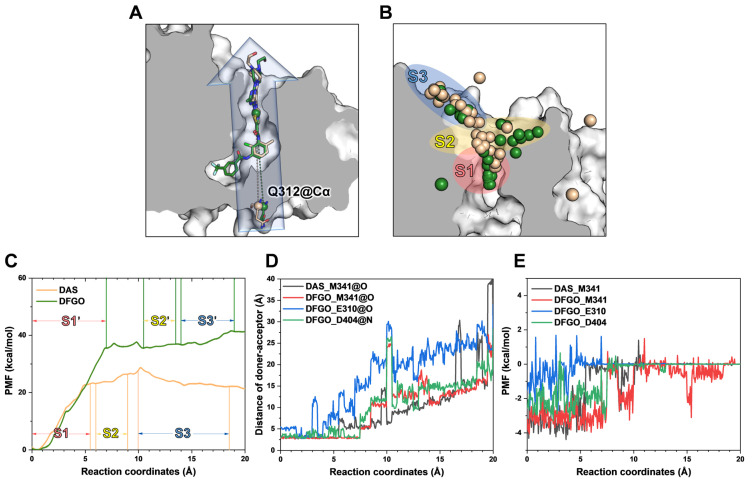
Umbrella sampling reveals the dissociation process of DAS and DFGO from c-Src: (**A**) Dissociation directory predicted by Caver 2.0. The wheat represents DAS, and green represents the DFGO system. (**B**) Dissociation trajectories of DAS and DFGO from c-Src. Conformations from the last 1 ns of trajectory were used to visualize the dissociation pathway. Each sphere represents the center of mass of ligands. Both DAS and DFGO moved from the ATP binding pocket (S1, pink shade) into the bulk solvent through S2 (yellow shade) and S3 stage (blue shade). (**C**) Energy profiles of DAS and DFGO during the various stages of dissociation. (**D**) In the umbrella sampling, the distances between the initial hydrogen bond donors and acceptors for M341, E310, and D404 with respect to DAS and DFGO are plotted as a function of the reaction coordinate. (**E**) The energy changes between DAS and DFGO with M341, E310, and D404 during the dissociation process.

To identify the structural features governing the dissociation of DAS and DFGO from c-Src, we performed umbrella sampling (US) for both systems (Figure 3A). During the entire US process, the system reached convergence at 15 ns (Figure S2). The potential of mean force (PMF) analysis showed that DFGO exhibits a higher dissociation barrier than DAS (Figure 3C), indicating that greater bias potential is required to dissociate DFGO. This finding aligns with the observed longer residence time of DFGO. This finding is consistent with the research of Tian et al., which indicates that increasing the energy barrier can extend the residence time of compounds [40]. The dissociation process of ligands was divided into three stages: S1 (pink shade), S2 (yellow shade), and S3 (blue shade) (Figure 3B). During the S1 stage, the ligand is located in the ATP binding pocket, while S2 and S3 represent subsequent dissociation stages. At the binding site, M341 formed hydrogen bonds with both DAS and DFGO, whereas D404 and E310 formed hydrogen bonds exclusively with DFGO (Figure 2A).

Subsequently, we monitored the distances and energetic contributions of hydrogen bonds formed between M341, D310, D404, and the ligand throughout the dissociation process. In the DAS systems, the distance between M341 and DAS increased sharply at a reaction coordinate (RC) of approximately 5.4 Å (Figure 3D, black line), coinciding with a significant drop in energy contribution (Figure 3E, black line), indicative of hydrogen bond cleavage. The potential of mean force (PMF) analysis corroborated that the ligand was transitioning between S1 and S2 at this point. In the DFGO system, a sharp increase in the distance between M341 and DFGO occurred at an RC of approximately 7.4 Å (Figure 3D, red line), accompanied by a concurrent decrease in energy contribution (Figure 3E, red line), marking hydrogen bond cleavage. Compared to DAS, the hydrogen bond between DFGO and M341 exhibited greater stability during dissociation, likely due to DFGO’s enhanced occupancy of the allosteric site, which contributes to increased binding stability. The hydrogen bond between DFGO and E310 displayed substantial fluctuations in the atomic distance (Figure 3D, blue line) and relatively minor or even positive energy contributions (Figure 3E, blue line), indicating an unstable interaction. Conversely, the hydrogen bond between DFGO and D404 showed less fluctuation in both distance and energy contribution during dissociation compared to the bond with E310 (Figure 3D,E, green line), but it is far less stable than the bond with M314. 

**Figure 4:**
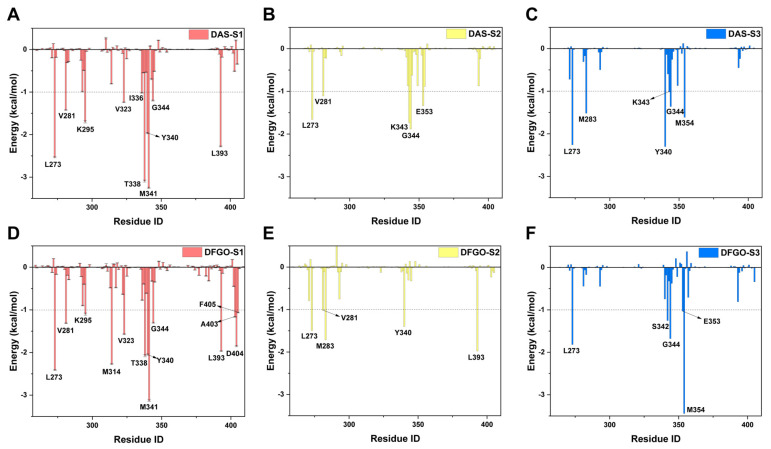
Energy contribution of key residues during the three stages (S1, S2, and S3) of DAS and DFGO dissociation. Residues with energy contribution less than −0.1 kcal/mol are regarded as key residues. Panels (**A**–**C**) illustrate the energy contributions of residues during the dissociation stages of DAS, while Panels (**D**–**F**) depict those for DFGO.

**Figure 5:**
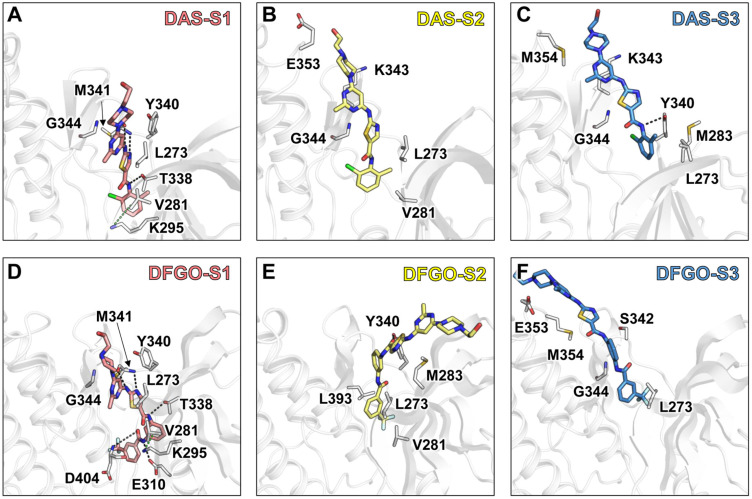
Interaction mode of representative conformations during the three stages of DAS and DFGO dissociations. Black dashed lines represent hydrogen bonds, while green dashed lines represent π–π stacking. Panels (**A**–**C**) correspond to the three stages of DAS, whereas Panels (**D**–**F**) correspond to the three stages of dasatinib.

Further analysis of residue energy contributions for representative conformations during the dissociation process reveals that the hydrogen bonds involving M341 and D404 are pivotal in stabilizing ligand binding to c-Src when the ligand is located within the binding pocket (S1 stage, Figure 4A,D). For example, DAS formed hydrogen bonds with M341 and T338 (Figure 5A), whereas DFGO not only formed hydrogen bonds with M341 and T338 but also with D404 and E310 (Figure 5D). As the ligand begins to dissociate, these hydrogen bonds break, and hydrophobic interactions become the predominant factors maintaining the ligand and c-Src binding. During the S2 stage, L273, V281, and G344 are crucial for DAS’s binding (Figure 4B and Figure 5B), whereas L273, V281, M283, and L393 are essential for DFGO’s binding (Figure 4E and Figure 5E). In the final dissociation stage (S3), hydrophobic residues such as L273, Y340, G344, and M354 make the greatest contribution to binding energy (Figure 4C,F). The binding modes analysis during dissociation indicates that the hydrophobic interactions formed by the trifluoromethylbenzyl group of DFGO largely account for the higher dissociation barrier of DFGO compared to DAS, underscoring the critical role of the trifluoromethylbenzyl group in stabilizing the DFGO and c-Src complex.

### 2.3. Compound Optimization

**Figure 6:**
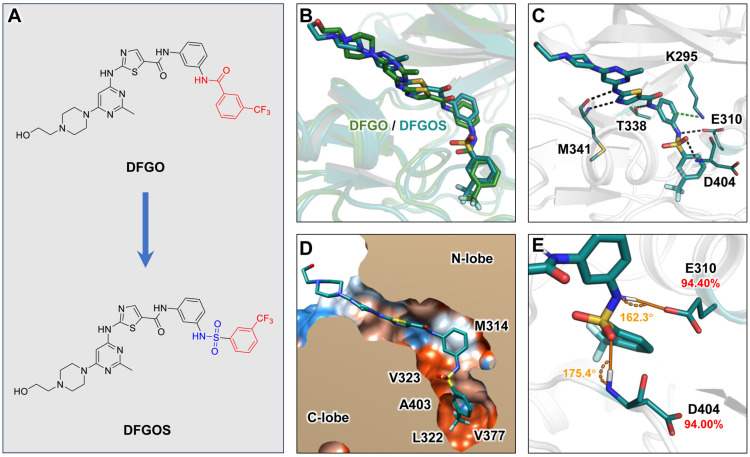
Structural characterization of the interaction between DFGOS and c-Src: (**A**) Structural design of DFGOS. (**B**) Superimposition of the binding poses of DFGO and DFGOS with c-Src. (**C**) Interaction mode of DFGOS with c-Src. Black and green dashed lines represent hydrogen bonds and π–π stacking, respectively. (**D**) Hydrophobic interaction map of DFGO. Polar regions are depicted in blue, and hydrophobic regions are shown in orange. (**E**) Angles and occupancy of hydrogen bonds formed between E310, D404, and DFGOS, with the donor–acceptor distance of the hydrogen bond between DFGO and D404 measured at 3.1 Å.

The above analyses indicate that the prolonged residence time of DFGO is primarily attributed to its trifluorobenzoyl amide group, which forms hydrogen bonds with D404 and E310 alongside hydrophobic interactions with residues such as L322, V377, and M354 within the binding pocket and along the dissociation pathway. However, these hydrogen bonds are unstable due to suboptimal bond angles. To address this instability, we substituted the amide group with a sulfonamide, a known bioisostere of the amide [41], while retaining the trifluoromethylbenzene moiety of DFGO to enhance stability both within the binding pocket and during dissociation. This modification resulted in the development of a new compound, DFGOS, designed to optimize the hydrogen bond angles and improve stability (Figure 6A). The binding mode of DFGOS closely resembles that of DFGO (Figure 6B). It can form hydrogen bonds with M341, E310, and D404 (Figure 6C). Additionally, its trifluoromethylbenzyl group is also located in the hydrophobic allosteric pocket (Figure 6D). MD simulations indicated that DFGOS exhibited an improved binding affinity to the target (ΔG = −38.82 kcal/mol) compared to DFGO (ΔG = −30.33 kcal/mol), primarily due to a significant increase in electrostatic interactions. Notably, the energy contribution of residue E310 shifted from being detrimental (positive value) to favorable (negative value) (Table 1). The occupancy of hydrogen bonds formed between DFGOS and residues E310 and D404 increased significantly, from 63.87% and 54.52% with DFGO to 94.40% and 94.00% with DFGOS, respectively (Figure 6E), indicating enhanced stability. In summary, DFGOS retains the beneficial structural feature of the trifluoromethylbenzene group while addressing the issue of hydrogen bond instability. This enhancement leads to higher hydrogen bond occupancy and increased binding free energy, suggesting that DFGOS will likely exhibit a longer residence time in c-Src than DFGO.

## 3. Materials and Methods

### 3.1. Conventional Molecular Dynamics (cMD) Simulations

The initial 3D coordinates of the c-Src–dasatinib and c-Src–DFGO complexes were obtained from the RCSB Protein Data Bank (PDB ID: 3G5D and 4YBJ, respectively). The E310A–dasatinib complex was constructed by mutating E310 to A310 using the Mutagenesis Wizard module in PyMOL (2020. http://www.pymol.org/pymol accessed 8 January 2024). For the c-Src–DFGOS complex, DFGOS was docked into the DFGO binding site using LeDock (v1.0) software [42].

All molecular dynamics simulations were performed using the Amber 20 software [43]. Each system was solvated in a truncated rectangular box of TIP3P water molecules [44], with at least 12 Å around the solute. To replicate physiological ionic strength, 0.15 M NaCl was added. Ligand structures were optimized using Gaussian 09 software [45] at the Hartree–Fock level with the 6–31G* basis set, and atomic partial charges were determined via the restrained electrostatic potential (RESP) fitting method [46]. Small molecules were described using the General Amber Force Field (GAFF) [47], while the receptor was parameterized with the AMBER ff19SB force field [48].

For the simulation setup, the system was initially minimized using the steepest descent method, followed by the conjugate gradient method to eliminate any steric collision. The systems were then gradually heated from 0 to 300 K in the canonical (NVT) ensemble, with solute atoms constrained by 5.0 kcal·mol^−1^ Å^−2^ harmonic restraint force. This was followed by a five-step equilibration process in the isothermal isobaric (NPT) ensemble, during which the restraint force on solute atoms was progressively reduced from 5.0 to 0 kcal·mol^−1^ Å^−2^. Subsequently, 1000 ns simulations were conducted for the c-Src–dasatinib and c-Src–DFGO systems, and 100 ns simulations for the E310A–dasatinib and c-Src–DFGOS systems, all within the NPT ensemble at 300 K and 1 atm, without any restraints. The time step was set to 2 fs. The SHAKE algorithm [49] was employed to constrain bond lengths involving hydrogen atoms, and long–range electrostatics interactions were calculated using the Particle Mesh Ewald (PME) method [50]. All simulated systems were run in parallel three times.

### 3.2. Umbrella Sampling (US)

Umbrella sampling is an enhanced sampling method. We followed procedures as described previously [28]. Briefly, the reaction coordinate (RC) for the US simulations was defined along the ATP binding pocket direction, as determined by Caver 2.0 [51]. Specifically, the RC was the distance between the center of mass of the ligand and the Cα atom of Q312 in DAS and DFGO systems. The umbrella sampling consisted of 41 windows, with the distance between the ligand and the binding site increased by 0.5 Å in each window, using harmonic restraints with a force constant of 5 kcal·mol^−1^ Å^−2^. To obtain the potential of mean force (PMF) along the RC, we applied the weighted histogram analysis method (WHAM) [52], which normalizes the skewed probability distribution. Each cycle of the US simulation, defined as a 41 ns US run, partitioned the RC into 2000 bins for the WHAM calculation, with a total of 615 ns performed for each system.

### 3.3. Simulation Analysis

To uncover the binding mechanism of ligands with different residence time to c-Src, trajectories were analyzed from both structural and energy perspectives using the CPPTRAJ program [53]. Conformational fluctuations and structural stability were assessed by monitoring the root-mean-square deviation (RMSD) of backbone atoms relative to the initial structure. A clustering analysis was performed using a hierarchical clustering algorithm to monitor the interaction between small molecules and c-Src. Principal component analysis (PCA) was conducted to investigate conformational space changes. For PCA, the covariance matrix was calculated using the RMSD value of the heavy atoms of DFGO, D404 and E310 from the last 100 ns trajectories, then diagonalized to obtain the principal components eigenvectors. The first two principal components were subsequently used as reaction coordinates to construct the free energy landscape.

### 3.4. MM/GBSA Calculation

The binding free energy of c-Src with ligands was calculated using the MM/GBSA method [54,55]. The detailed calculation process is the same as in our previous work [28,56]. Briefly, the last 10% of the trajectories for all studied systems were extracted every ten frames for energy calculation. The binding free energy ∆G is defined as the sum of the enthalpy term (∆H) and entropy term (–∆S) contribution. The enthalpy change ∆H of the system is composed of the enthalpy changes in the gas phase upon complex formation (ΔE_gas_) and the solvated free energy contribution (ΔG_sol_), expressed as ∆H = ΔE_gas_ + ΔG_sol_. The ΔE_gas_ is the sum of the internal interaction from bonds, angels and torsions, electrostatic interactions (ΔE_ele_), and van der Walls interaction energy (ΔE_vdw_). The solvation-free energy is composed of the polar and the nonpolar contributions: ΔG_sol_ = ΔG_GB_ + ΔG_np_. To identify the key residues in the binding process between ligands and c-Src, per-residue free energy decomposition was performed using the MM/GBSA method. The free energy decomposition included as-phase and solvation energy calculations, excluding entropy contribution.

## 4. Conclusions

This study utilized molecular dynamics simulation to elucidate the structural characteristics of c-Src binding with antagonists of varying residence times. The antagonist DFGO, which exhibits an extended residence time, prolongs its interaction with c-Src by occupying an allosteric site through its trifluorobenzamide group, establishing hydrogen bonds with D404 and E310, and engaging in hydrophobic interactions with such as L322 and V377. However, these hydrogen bonds formed with D404 and E310 were found to be unstable. By substituting the amide group in DFGO with a sulfonamide, the derivative compound, DFOGS, achieved more stable hydrogen bonds with D404 and E310, thereby enhancing the overall stability of the complex. For the design of long residence time c-Src antagonists, incorporating hydrophobic groups to occupy the allosteric pocket and stabilizing the interaction through both hydrogen bonding and hydrophobic interactions could effectively prevent ligand dissociation and prolong binding duration with the target. These findings provide a valuable framework for the rational design of long residence time c-Src antagonists.

## Figures and Tables

**Table 1:** Calculated binding free energies (∆G, kcal/mol) for DAS, DFGO, DFGO-E310A, and DFGOS with c-Src, along with the energy contribution of E310.

Contributions	Systems			
	DAS	DFGO	DFGO-E310A	DFGOS
ΔE_vdw_	−59.32	−74.32	−71.68	−78.31
ΔE_ele_	−20.19	−88.05	−69.07	−97.64
ΔG_GB_	31.79	109.35	91.28	111.87
ΔG_np_	−6.39	−9.12	−8.92	−9.37
ΔE_gas_	−79.52	−162.36	−140.74	−175.95
ΔG_sol_	25.40	100.23	82.36	102.50
∆H	−54.12	−62.14	−58.39	−73.45
−∆S	24.52	31.81	25.69	34.63
∆G	−29.60	−30.33	32.70	−38.82
E310	-	0.09	−0.34	−1.08

## Data Availability

Data are contained within the article and Supplementary Materials.

## Supplementary Materials

The following supporting information can be downloaded at: https://www.mdpi.com/article/10.3390/ijms251910477/s1.
