# Supplementary material for: Structural Basis for Long Residence Time c-Src Antagonist: Insights from Molecular Dynamics Simulations
Source: Int J Mol Sci. 2024 Sep 28;25(19):10477. doi: 10.3390/ijms251910477 (PMC11476938; doi:10.3390/ijms251910477)
Supplement: Supplementary file 1 [file ijms-25-10477-s001.zip › ijms-3204867-supplementary.pdf]

Supporting Information

Table S1. Binding free energies (ΔG, kcal/mol) of DFGO interacting with c-Src across three parallel trajectories, including the energy contribution of E310. The binding free energy ΔG is defined as the sum of the enthalpy term (ΔH) and entropy term (-ΔS) contribution. The enthalpy change ΔH of the system is composed of the enthalpy changes in the gas-phase upon complex formation (ΔE_{gas}) and the solvated free energy contribution (ΔG_{sol}), expressed as $\Delta H = \Delta E_{gas} + \Delta G_{sol}$. The ΔE_{gas} is the sum of the internal interaction from bonds, angels and torsions, electrostatic interactions (ΔE_{ele}) and van der Walls interaction energy (ΔE_{vdw}). The solvation free energy is composed of the polar and the nonpolar contributions: $\Delta G_{sol} = \Delta G_{GB} + \Delta G_{np}$.

Contributions	Systems		
	DFGO_run1	DFGO_run2	DFGO_run3
ΔE_{vdw}	-74.32	-73.18	-71.42
ΔE_{ele}	-88.05	-91.31	-84.51
ΔG_{GB}	109.35	109.79	103.47
ΔG_{np}	-9.12	-8.97	-8.66
ΔE_{gas}	-162.36	-164.48	-155.74
ΔG_{sol}	100.23	100.81	94.80
ΔH	-62.14	-63.67	-60.94
-ΔS	31.81	30.85	32.28
ΔG	-30.33	-32.82	-28.66
E310	0.09	0.05	0.12

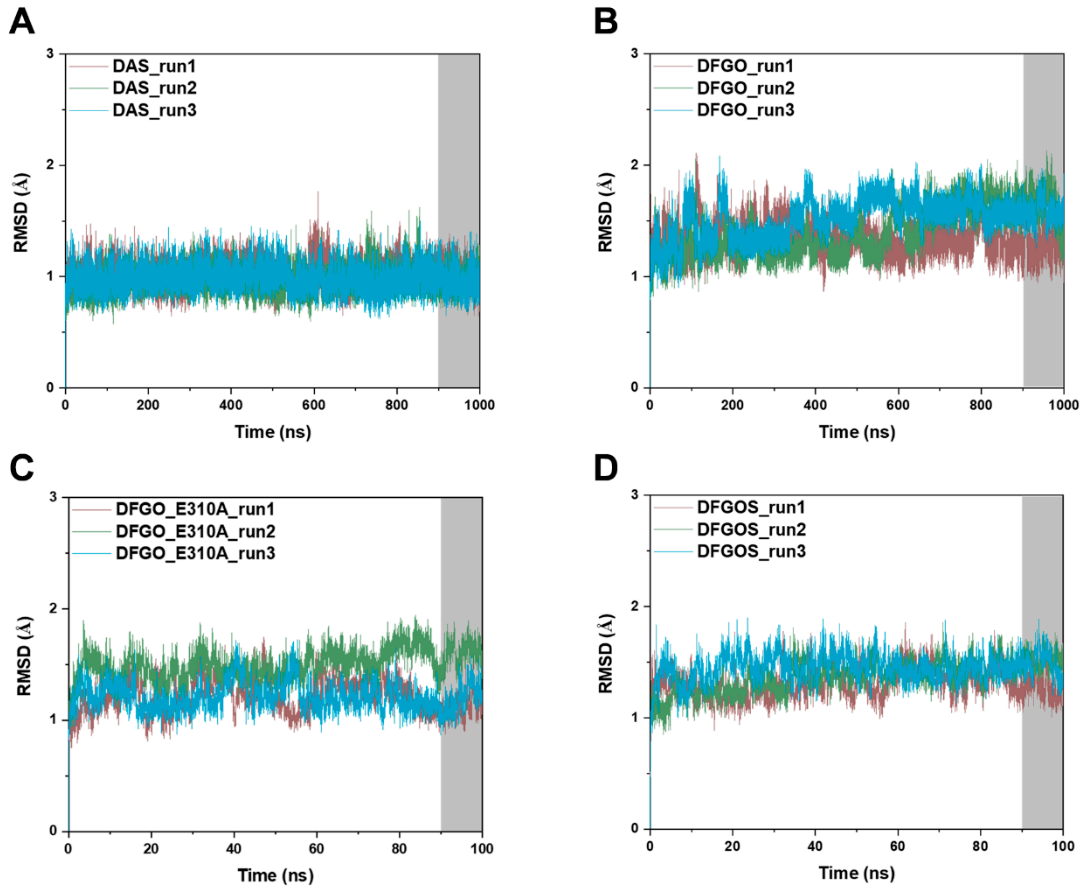

Figure S1. Time evolution of the root-mean-square deviation (RMSD) for the DAS, DFGO, DFGO_E310A, and DAFOS complexes with c-Src was evaluated across three independent replicates for each system. Convergence was reached in the final 10% of the simulation trajectories, and analyses were performed based on these converged trajectories.

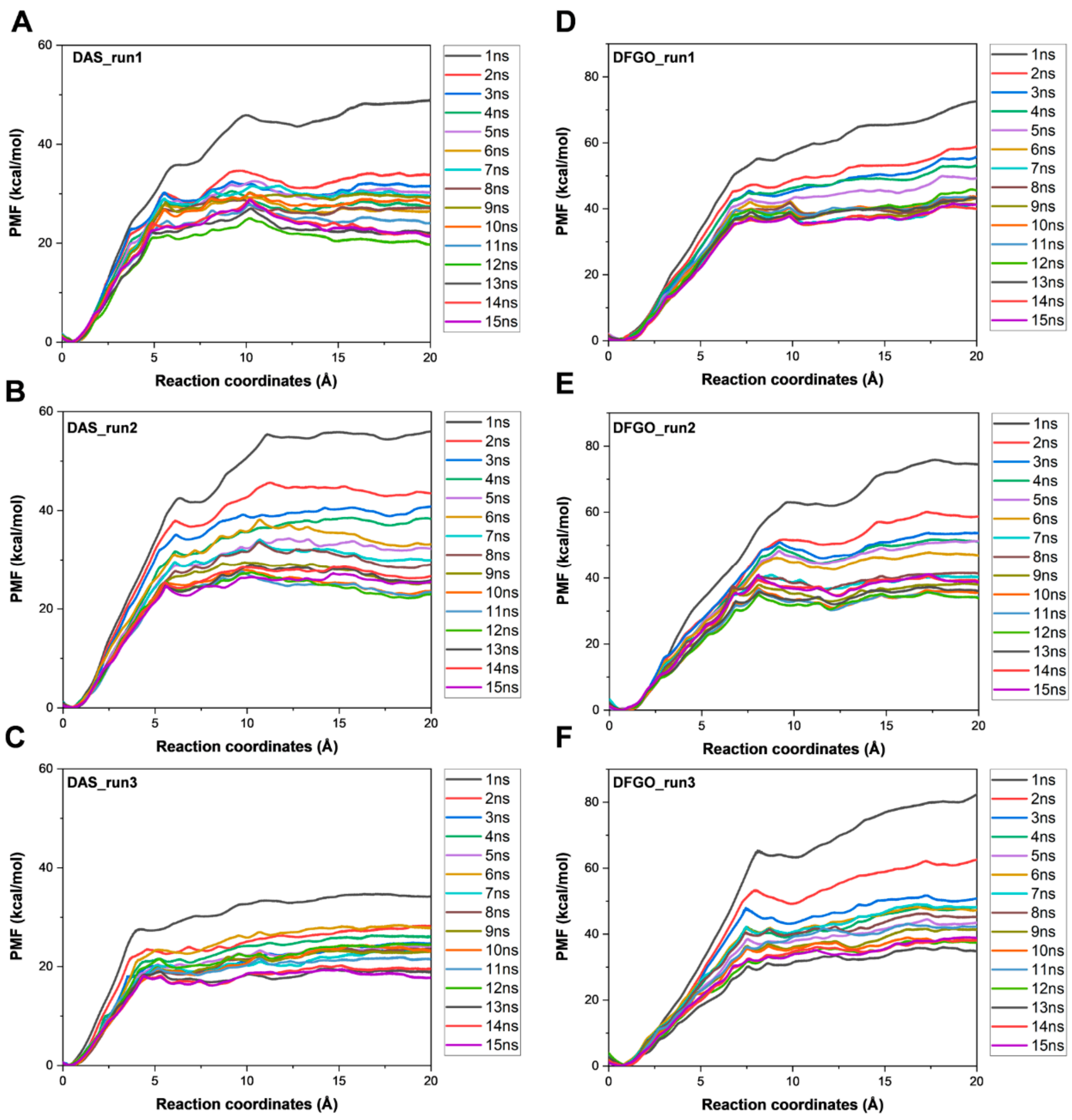

Figure S2. The potential of mean force (PMF) profiles for DAS and DFGO were calculated based on umbrella sampling trajectories. The umbrella sampling included a total of 41 windows, each running for 1 ns. Energy convergence was achieved for all systems by the 15th iteration. Structural and energy analyses were conducted based on the trajectories obtained from the final iteration. Each system was independently repeated three times using the umbrella sampling method.
